# Uptake of robot-assisted colon cancer surgery in the Netherlands

**DOI:** 10.1007/s00464-023-10383-5

**Published:** 2023-08-29

**Authors:** Marlou F. M. Sterk, Rogier M. P. H. Crolla, Mareille Verseveld, Jan Willem T. Dekker, George P. van der Schelling, Cornelis Verhoef, Pim B. Olthof

**Affiliations:** 1 Department of Surgery, Amphia Hospital, Breda, The Netherlands; 2 Department of Surgery, Franciscus Gasthuis & Vlietland, Rotterdam, The Netherlands; 3 Department of Surgery, Reinier de Graaf Gasthuis, Delft, The Netherlands; 4 Department of Surgery, Erasmus MC Cancer Institute, Dr. Molewaterplein 40, 3015GD Rotterdam, The Netherlands

**Keywords:** Colon cancer, Robotic surgery, Colectomy, Robot-assisted colectomy, Minimally invasive

## Abstract

**Background:**

The robot-assisted approach is now often used for rectal cancer surgery, but its use in colon cancer surgery is less well defined. This study aims to compare the outcomes of robotic-assisted colon cancer surgery to conventional laparoscopy in the Netherlands.

**Methods:**

Data on all patients who underwent surgery for colon cancer from 2018 to 2020 were collected from the Dutch Colorectal Audit. All complications, readmissions, and deaths within 90 days after surgery were recorded along with conversion rate, margin and harvested nodes. Groups were stratified according to the robot-assisted and laparoscopic approach.

**Results:**

In total, 18,886 patients were included in the analyses. The operative approach was open in 15.2%, laparoscopic in 78.9% and robot-assisted in 5.9%. The proportion of robot-assisted surgery increased from 4.7% in 2018 to 6.9% in 2020. There were no notable differences in outcomes between the robot-assisted and laparoscopic approach for Elective cT1-3M0 right, left, and sigmoid colectomy. Only conversion rate was consistently lower in the robotic group. (4.6% versus 8.8%, 4.6% versus 11.6%, and 1.6 versus 5.9%, respectively).

**Conclusions:**

This nationwide study on surgery for colon cancer shows there is a gradual but slow adoption of robotic surgery for colon cancer up to 6.9% in 2020. When comparing the outcomes of right, left, and sigmoid colectomy, clinical outcomes were similar between the robotic and laparoscopic approach. However, conversion rate is consistently lower in the robotic procedures.

Robot-assisted surgery has several potential benefits over conventional laparoscopy. These include the stable 3D images controlled by the surgeon with better visualization of small details and the angulated instruments that allow more precise dissection in areas otherwise difficult to access [1]. In addition, the position of the surgeon in the console allows better ergonomics [2].

These features make robot-assisted surgery especially helpful in confined spaces such as the pelvis [3]. Consistently, rectal resections are among the most frequently performed robot-assisted procedures [4].Numerous studies have shown that the implementation of robot-assisted rectal resection was safe with similar short-term morbidity and oncological outcomes compared to conventional laparoscopy. However, there is no clear evidence that the outcomes of robot-assisted rectal resection are superior to conventional laparoscopy besides a lower conversion rate [5, 6]. Nevertheless, the proportion of rectal resections performed robotically is increasing [4].

In line with the implementation of robot-assisted surgery in rectal cancer, also colon resections are increasingly performed with the robot [7]. Although the prospected benefits of robot-assisted surgery might be less than those in rectal cancer, sigmoid resection has a lot of similarities to rectal cancer surgery. In addition, the excellent visualization with the robotic platform might help with complete mesocolic excision in right colectomy and mobilization of the splenic flexure in left colectomy, which can be considered as technically challenging [8, 9]. While the number of reports is increasing, data on robot-assisted colon resection need to be better defined, the series are mostly small and from experienced robotic surgeons [10, 11]. Outcomes from such series might not apply to less experienced centers and nationwide data might better reflect actual daily practice.

This study aimed to evaluate the implementation of robot-assisted colon cancer surgery in the Netherlands using data of the National colon cancer surgery audit. The study analyzed the proportion of procedures performed robot-assisted and the associated outcomes compared to conventional laparoscopy.

## Methods

All patients who underwent surgery for colon cancer between January 1th 2018 and December 31th 2020 were included in this study. All data were obtained from the mandatory Dutch Colorectal Audit (DCRA), in which all Dutch hospitals are required to enter their data. The study protocol was approved by the scientific committee of the DCRA. Separate ethical approval was not required under Dutch law, due to the anonymous extraction of data.

All patients who underwent a resection for colon cancer were included for all stages, with a subgroup analysis for those staged cT1-3M0. Patients who underwent resection for rectal cancer were excluded. Patients who were classified to have colon cancer, but who underwent total mesorectal excision, abdominal perineal resection, or proctocolectomy were excluded, as these patients are likely to have had rectal cancer instead. Patients with a local excision, colonic wedge resection, unspecified procedure, or unknown operative approach were also excluded. All remaining patients were included. The main analyses focused on the largest procedure groups that included right hemicolectomy, left hemicolectomy, and sigmoid resections. All robotic procedures were performed using the DaVinci robotic systems.

All complications, readmissions, and deaths within 90 days after surgery were recorded. Re-interventions included all radiological, endoscopic, and surgical interventions within 90 days after surgery with or without general anesthesia.

All categorical data are presented as numbers and percentages. Differences between continuous variables were tested using Fisher’s exact tests. All continuous data are presented as median with inter-quartile ranges (IQR) and differences were tested using Mann–Whitney *U*-tests. Multivariable analysis was performed using logistic regression. All variables with a p value of 0.1 or lower were included in the multivariable analysis with backward selection. Statistical analysis were performed using SPSS (version 24.0, IBM Inc, Chicago, IL). Graphs were generated using GraphPad Prism (Graphpad Inc., La Jolla, CA).

## Results

In total 20,328 patients underwent surgery for colon cancer in the study period. Out of all procedures, 545 were classified as total mesorectal excision, abdominal perineal resection, or proctocolectomy and these were excluded. Other exclusions were 259 patients who underwent an unspecified procedure, 13 patients who underwent local excision and 18 patients who underwent wedge colonic resection. The operative approach was unspecified for 607 patients, these were also excluded. The remaining 18,886 patients were included in the analyses.

**Fig. 1:**
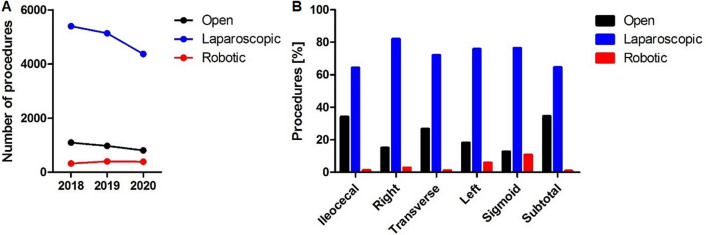
**A** Number of open, laparoscopic, and robotic resection for colon cancer per year in the Netherlands. **B** The proportion of open, laparoscopic, and robotic approach per specified procedure

The operative approach was open in 15.2%, laparoscopic in 78.9% and robot-assisted in 5.9%. The proportion of robot-assisted surgery increased from 4.7% in 2018 to 6.9% in 2020 and while the absolute number of open and laparoscopic cases decreased over the studied years, the number of robotic cases increased (Fig. 1A). The proportion of robotic surgery was highest for sigmoid resections (10.8%) followed by left hemicolectomies (5.9%) and right hemicolectomies (2.9%). For ileocecal resection, transverse colon resection, and subtotal colectomy, the proportion of robotic surgery was 0.9 to 1.4% (Fig. 1B).

Patient, disease, and operative characteristics were similar for robotic and laparoscopic right colectomy (Table 1). The only difference was more elective cases in the robotic group (98.6 versus 94.5%, *P* = 0.001). R1 resection rate and adverse outcome rates were similar between the approaches. The median number of harvested lymph nodes was higher in the robotic group (23 (18–31) versus 21 (16–29), *P* = 0.003). Although median hospital stay was 4 days in both groups, there was a statistically significant difference in favor of the robotic group [4 (3–6) versus 4 (3–7) days, *P* = 0.018]. Conversion rate was lower in the robotic group (5.0 versus 11.1%, *P* < 0.001). After exclusion of non-elective, cT4, and cM1 cases, the conversion rate remained lower in the robotic group, and the median number of harvested nodes remained higher in the robotic group. Other outcomes including hospital stay were similar.

**Table 1:** Comparison of robotic and laparoscopic right colectomy in all and elective cT1-3M0 cases

Right hemicolectomy	Robotic (*n* = 281)	Laparoscopic (*n* = 7821)	*P* value
Age, median (IQR)	73 (67–80)	73 (66–79)	0.975
Male sex, n (%)	136 (48.4)	3557 (45.5)	0.361
ASA III/IV, n (%)	92 (32.7)	2904 (37.1)	0.148
BMI, kg/m^2^, median (IQR)	25.7 (23.4–28.9)	26.0 (23.4–29.1)	0.673
cT4, n (%)	8 (2.8)	429 (5.5)	0.059
cM1, n (%)	15 (5.3)	355 (4.5)	0.469
Neoadjuvant chemotherapy, n (%)	7 (2.5)	107 (1.4)	0.118
Elective surgery, n (%)	277 (98.6)	7388 (94.5)	0.001
Anastomosis, n (%)	277 (98.6)	7621 (97.4)	0.329
Additional local resection, n (%)	25 (8.9)	519 (6.6)	0.144
Synchronous metastasis resection, n (%)	7 (2.5)	106 (1.4)	0.115
Hospital stay, days, median (IQR)	4 (3–6)	4 (3–7)	0.018
R1 resection, n (%)	1 (0.4)	50 (0.6)	1.000
Number of harvested nodes, median (IQR)	23 (18–31)	21 (16–29)	0.003
Conversion, n (%)	14 (5.0)	868 (11.1)	< 0.001
Morbidity, n (%)	72 (25.6)	2042 (26.1)	0.890
Anastomotic leakage, n (%)	7 (2.5)	280 (3.6)	0.412
Reintervention rate, n (%)	15 (5.3)	585 (7.5)	0.202
90 days Readmission rate, n (%)	22 (7.8)	665 (8.5)	0.827
90 days mortality, n (%)	3 (1.1)	201 (2.6)	0.169

Right hemicolectomy—elective cT1-3M0	Robotic (*n* = 257)	Laparoscopic (*n* = 6774)	*P* value
Age, median (IQR)	73 (67–79)	73 (67–79)	0.904
Male sex, n (%)	127 (49.4)	3049 (45.1)	0.180
ASA II/IV, n (%)	85 (33.1)	2503 (37.0)	0.211
BMI, kg/m^2^, median (IQR)	25.7 (23.6–29.1)	26.1 (23.6–29.2)	0.769
Neoadjuvant chemotherapy, n (%)	3 (1.2)	30 (0.4)	0.118
Anastomosis, n (%)	253 (98.4)	6626 (97.8)	0.762
Additional local resection, n (%)	18 (7.0)	292 (4.3)	0.044
Synchronous metastasis resection, n (%)	1 (0.4)	30 (0.4)	1.000
Hospital stay, days, median (IQR)	4 (3–6)	4 (3–7)	0.109
R1 resection, n (%)	0 (0)	23 (0.3)	1.000
Number of harvested nodes, median (IQR)	23 (17–30)	21 (16–29)	0.006
Conversion, n (%)	12 (4.6)	602 (8.8)	0.018
Morbidity, n (%)	69 (26.8)	1696 (25.1)	0.510
Anastomotic leakage, n (%)	7 (2.7)	239 (3.5)	0.605
Reintervention rate, n (%)	15 (5.8)	484 (7.1)	0.535
90 days Readmission rate, n (%)	21 (8.2)	552 (8.2)	1.000
90 days mortality, n (%)	3 (1.2)	141 (2.1)	0.496

All patient, disease, and operative characteristics were similar for robotic and laparoscopic left colectomy (Table 2). After exclusion of non-elective, cT4, and cM1 cases, the conversion rate was lower in robotic cases (4.6 versus 11.6%, *P* = 0.025). The median number of harvested nodes was lower in the robotic group [15 (12–21) versus 16 (12–23), *P* = 0.038]. All other characteristics and outcomes were similar.

**Table 2:** Comparison of robotic and laparoscopic left colectomy in all and elective cT1-3M0 cases

Left hemicolectomy	Robotic (*n* = 130)	Laparoscopic (*n* = 1663)	*P* value
Age, median (IQR)	71 (61–76)	70 (62–76)	0.884
Male sex, n (%)	70 (54.7)	913 (55.0)	1.000
ASA III/IV, n (%)	41 (31.5)	514 (30.9)	0.922
BMI, kg/m^2^, median (IQR)	26.7 (24.4–29.6)	26.2 (23.5–29.4)	0.319
cT4, n (%)	4 (3.1)	87 (5.5)	0.404
cM1, n (%)	5 (3.8)	98 (5.9)	0.434
Neoadjuvant chemotherapy, n (%)	2 (1.5)	23 (1.4)	0.702
Elective surgery, n (%)	115 (88.5)	1533 (92.2)	0.134
Anastomosis, n (%)	123 (94.6)	1538 (92.5)	0.485
Additional local resection, n (%)	12 (9.2)	113 (6.8)	0.283
Synchronous metastasis resection, n (%)	–	30 (1.8)	0.164
Hospital stay, days, median (IQR)	5 (3–7)	4 (3–7)	0.672
R1 resection, n (%)	1 (0.8)	14 (0.8)	1.000
Number of harvested nodes, median (IQR)	16 (12–21)	17 (12–23)	0.139
Conversion, n (%)	11 (8.5)	229 (13.8)	0.107
Morbidity, n (%)	44 (33.8)	434 (26.1)	0.063
Anastomotic leakage, n (%)	6 (4.6)	90 (5.4)	0.841
Reintervention rate, n (%)	8 (6.2)	169 (10.2)	0.169
90 days Readmission rate, n (%)	16 (12.3)	144 (8.7)	0.153
90 days mortality, n (%)	2 (1.5)	33 (2.0)	1.000

Left hemicolectomy—elective cT1-3M0	Robotic (*n* = 108)	Laparoscopic (*n* = 1389)	*P* value
Age, median (IQR)	70 (59–74)	71 (63–76)	0.270
Male sex, n (%)	56 (51.9)	776 (55.9)	0.432
ASA II/IV, n (%)	33 (30.6)	414 (29.8)	0.912
BMI, kg/m^2^, median (IQR)	26.9 (25.6–30.0)	26.4 (23.8–29.6)	0.235
Neoadjuvant chemotherapy, n (%)	0 (0)	6 (0.4)	1.000
Anastomosis, n (%)	104 (96.3)	1305 (94.0)	0.400
Additional local resection, n (%)	6 (5.6)	59 (4.3)	0.464
Synchronous metastasis resection, n (%)	0 (0)	9 (0.6)	1.000
Hospital stay, days, median (IQR)	4 (3–6)	4 (3–7)	0.367
R1 resection, n (%)	1 (0.9)	10 (0.7)	0.562
Number of harvested nodes, median (IQR)	15 (12–21)	16 (12–23)	0.038
Conversion, n (%)	5 (4.6)	161 (11.6)	0.025
Morbidity, n (%)	33 (30.6)	354 (25.5)	0.255
Anastomotic leakage, n (%)	5 (4.6)	81 (5.8)	0.829
Reintervention rate, n (%)	7 (6.5)	144 (10.4)	0.245
90 days readmission rate, n (%)	15 (13.9)	117 (8.4)	0.075
90 days mortality, n (%)	1 (0.9)	22 (1.6)	1.000

The characteristics of robotic and laparoscopic sigmoid resections are presented in Table3. In the robotic group ASA scores of III and IV were less frequent (22.8 versus 26.4, *P* = 0.045). There was less metastatic disease (4.2 versus 7.0%, *P* = 0.005), less additional resections due to local tumor extent (3.5% versus 5.9%, *P* < 0.001) and more anastomoses (94.0% versus 89.2%, *P* < 0.001) in the robotic group. R1 resection rate was lower in the robotic group (0% versus 0.7%, *P* = 0.028) and the conversion rate was lower (2.3% versus 8.2%, *P* < 0.001). All other outcomes were similar between the groups. After exclusion of non-elective, cT4, and cM1 cases, the number of patients that underwent neoadjuvant chemotherapy was higher in the robotic group (2.1% versus 1.0%, *P* = 0.022), and the number of patients in whom an anastomosis was created was higher compared to conventional laparoscopy (95.2% versus 92.5%, *P* = 0.014). Conversion rate was lower in the robot-assisted group (1.6% versus 5.9%, *P* < 0.001), all other outcomes were similar.

**Table 3:** Comparison of robotic and laparoscopic sigmoid resection in all and elective cT1-3M0 cases

Sigmoid resection	Robotic (*n* = 685)	Laparoscopic (*n* = 4869)	*P* value
Age, median (IQR)	69 (59–75)	69 (59–76)	0.373
Male sex, n (%)	405 (59.1)	2886 (59.3)	0.934
ASA III/IV, n (%)	156 (22.8)	1285 (26.4)	0.045
BMI, kg/m^2^, median (IQR)	26.0 (23.7–28.7)	26.1 (23.6–29.1)	0.607
cT4, n (%)	24 (3.7)	248 (5.4)	0.073
cM1, n (%)	29 (4.2)	343 (7.0)	0.005
Neoadjuvant chemotherapy, n (%)	31 (4.5)	146 (3.0)	0.037
Elective surgery, n (%)	653 (95.3)	4559 (93.6)	0.089
Anastomosis, n (%)	644 (94.0)	4345 (89.2)	< 0.001
Additional local resection, n (%)	24 (3.5)	286 (5.9)	< 0.001
Synchronous metastasis resection, n (%)	8 (1.2)	90 (1.8)	0.276
Hospital stay, days, median (IQR)	4 (3–5)	4 (3–5)	0.107
R1 resection, n (%)	–	33 (0.7)	0.028
Number of harvested nodes, median (IQR)	17 (13–23)	17 (12–23)	0.229
Conversion, n (%)	16 (2.3)	398 (8.2)	< 0.001
Morbidity, n (%)	139 (20.3)	932 (19.1)	0.469
Anastomotic leakage, n (%)	29 (4.2)	218 (4.5)	0.843
Reintervention rate, n (%)	47 (6.9)	333 (6.8)	1.000
90 day Readmission rate, n (%)	37 (5.4)	346 (7.1)	0.107
90 day mortality, n (%)	5 (0.7)	65 (1.3)	0.205

Sigmoid resection—Elective cT1-3M0	Robotic (*n* = 609)	Laparoscopic (*n* = 4110)	*P* value
Age, median (IQR)	68 (59–75)	69 (59–76)	0.244
Male sex, n (%)	362 (59.4)	2449 (59.7)	0.930
ASA II/IV, n (%)	130 (21.3)	1027 (25.0)	0.055
BMI, kg/m^2^, median (IQR)	26.0 (23.7–29.0)	26.2 (23.7–29.3)	0.322
Neoadjuvant chemotherapy, n (%)	13 (2.1)	41 (1.0)	0.022
Anastomosis, n (%)	580 (95.2)	3802 (92.5)	0.014
Additional local resection, n (%)	12 (2.0)	122 (3.0)	0.101
Synchronous metastasis resection, n (%)	1 (0.2)	21 (0.5)	0.348
Hospital stay, days, median (IQR)	4 (3–5)	4 (3–5)	0.393
R1 resection, n (%)	0 (0)	18 (0.4)	0.158
Number of harvested nodes, median (IQR)	17 (13–23)	16 (12–23)	0.150
Conversion, n (%)	10 (1.6)	242 (5.9)	< 0.001
Morbidity, n (%)	118 (19.4)	721 (17.5)	0.281
Anastomotic leakage, n (%)	25 (4.1)	183 (4.5)	0.752
Reintervention rate, n (%)	38 (6.2)	269 (6.5)	0.860
90 day Readmission rate, n (%)	32 (5.3)	258 (6.3)	0.366
90 day mortality, n (%)	4 (0.7)	37 (0.9)	0.814

Across all types of procedures in this cohort, conversion occurred in 10.1%. The conversion rate was 3.7% (41/1105) in the robot-assisted group and 10.6% (1580/14901) in the laparoscopic procedure group. When combined, conversion was associated with an increased morbidity (44.8% versus 21.9%, *P* < 0.001), reintervention (13.6% versus 7.0%, *P* < 0.001), readmission (12.2% versus 7.8%, *P* < 0.001), and mortality rate (5.0% versus 1.7%, *P* < 0.001). Median length of stay was also increased in case of conversion [7 (5–11) versus 4 (3–6) days, *P* < 0.001]. In a multivariable analysis for conversion, the robotic approach remained associated with a lower conversion rate compared to conventional laparoscopy (Table 4).

**Table 4:** Multivariable analysis for conversion during laparoscopic and robotic surgery for colon cancer

Right hemicolectomy	Odds ratio (95% CI)	*P* value	Odds ratio (95% CI)	*P* value
Age, continuous	1.02 (1.01–1.02)	< 0.001	1.01 (1.00–1.01)	0.003
Male sex	1.31 (1.17–1.45)	< 0.001	1.41 (1.25–1.58)	< 0.001
ASA III/IV	1.75 (1.57–1.95)	< 0.001	1.56 (1.38–1.76)	< 0.001
BMI, continuous	1.00 (1.00–1.01)	0.079		
Previous bowel surgery	2.94 (2.33–3.72)	< 0.001	2.97 (2.30–3.84)	< 0.001
Previous stoma	3.13 (2.04–4.79)	< 0.001	1.84 (1.15–2.94)	< 0.001
cT4, versus cT1-3	5.09 (4.30–6.02)	< 0.001	2.03 (1.67–2.47)	< 0.001
cM1	1.47 (1.19–1.79)	< 0.001		
Neoadjuvant chemotherapy	1.45 (1.04–2.01)	0.027		
Non-elective surgery	3.96 (3.38–4.63)	< 0.001	3.41 (2.88–4.04)	< 0.001
Additional local resection	6.05 (5.23–6.99)	< 0.001	4.57 (3.86–5.40)	< 0.001
Synchronous metastasis resection	1.19 (0.79–1.79)	0.400		
Procedure				
Right colectomy	Reference		Reference	
Left colectomy	1.29 (1.10–1.50)		1.29 (1.09–1.52)	
Sigmoid resection	0.67 (0.59–0.76)	0.001 < 0.001	0.73 (0.64–0.83)	< 0.001 < 0.001
Robotic approach	0.34 (0.25–0.46)	< 0.001	0.37 (0.27–0.52)	< 0.001

## Discussion

In this Nationwide study on 18,886 patients who underwent surgery for colon cancer, 5.9% of the procedures were performed robotically with a modest increase from 4.7% in 2018 to 6.9% in 2020. Overall, most characteristics and outcomes were similar when comparing the robotic to the laparoscopic approach. The conversion rate was consistently lower with the robotic approach for right hemicolectomy, left hemicolectomy and sigmoid resections.

Minimally invasive surgery has become the standard approach for colon cancer resections. The percentage of minimally invasive procedure for colon cancers varies per year, country, and cohort characteristics and ranges from 30 to 90% [12–15]. After the initial caution warranted by randomized trials on laparoscopic colon resection regarding oncological outcomes [16], there is now sufficient evidence that laparoscopic surgery is safe and effective [17–21]. The laparoscopic approach is associated with less morbidity and shorter hospital stay compared to open surgery [19, 20].

The robotic approach has several advantages over conventional laparoscopy that are especially advantageous in the confined space of the pelvis. While the benefit of the robotic approach in colectomy may be less clear, the use of the robotic approach is increasing for colon resections [7]. The robotic system allows better visualization, with a stable and 3D view. Although colon cancer surgery is less confined to one abdominal region, the enhanced visualization might help with more complex tasks such as complete mesocolic excision of mobilization of the splenic flexure. Using the articulating instruments and stable camera positioning these tasks might be more easy to achieve. However, the actual place of robotic surgery in colon cancer surgery is not yet defined. While almost 11% of cases were performed robotically in an American study on 191,292 patients, 45% of cases were still performed open in this study dating up to 2016. In the Netherlands, the adoption of laparoscopic surgery is much higher, yet only 6.9% of colon cancer procedures were performed using the robotic approach. Maybe the vast experience in laparoscopic surgery limits surgeons in their perceived additional benefits of other minimally invasive techniques, such as robot-assisted procedures. This could form a threshold to engage a new learning curve.

Most data show similar learning curves for robotic and laparoscopic colon surgery, while the learning curve for right colectomy might be shorter for the robotic approach [22]. However, data also show that the robotic approach can be faster for mesorectal excision and more complex steps such as knot tying [22, 23]. A longer operative time using the robotic approach is a frequent criticism, but is likely to decline over time with increasing experience. However, the learning curve based on operative time might be a poor endpoint since more complex cases are usually taken up with increasing experience, which may counter the reduction in operative time [24].

The only randomized trial on robotic versus laparoscopic right colectomy did not show a difference in outcomes and concluded the increased costs associated with robotic surgery is not justified [25]. In that report there were no conversions, yet in a later meta–analysis a lower conversion rate was reported in robotic right colectomy, without differences in morbidity and mortality, at the expense of longer operative time and higher costs [10]. Results were similar in a meta-analysis on left colectomy [26]. In the present study we confirm the lower conversion rate associated with robotic procedures, with similar other outcomes compared to laparoscopic surgery. Conversion was associated with worse clinical outcomes. While conversion can be reactive to adverse events or due to more advances disease, the lower rate in robotic procedures suggests with the gradual adoption and increasing experience the robotic platform might be able to improve short-term clinical outcomes.

Most studies on surgery for colon cancer report increased costs associated with robotic surgery, compared to conventional laparoscopy, as is reported for most robot-assisted procedures [10, 27]. The additional costs associated with robotic procedures should be balanced by reduced morbidity and length of stay [28]. Although data on cost-effectiveness were not available in this study, the only difference in outcomes was the lower conversion rate associated with robotic procedures. Therefore it is unlikely, the robotic approach outperformed the laparoscopic approach in this study.

The current study has several limitations mostly related to retrospective design. However, this nationwide study included all resections for colon cancer in the Netherlands. Data on individual centers were not available, nor was center or surgeon volume. These variables might be related to the outcomes of robotic procedures. Additional confounders that indicate patient selection operated using the robotic platform could be present. The clinical and operative protocol were not standardized, because this is a nationwide audit study, which might have influenced the results.

In conclusion, this nationwide study on surgery for colon cancer shows there is a gradual but slow adoption of robotic surgery for colon cancer up to 6.9% in 2020. When comparing the outcomes of right, left, and sigmoid colectomy, clinical outcomes were similar between the robotic and laparoscopic approach. However, conversion rate is consistently lower in the robotic procedures.
